# Skeletal Motor Unit Recruitment During Periodic Auditory Cueing: A Simultaneous Behavioral and Motor Unit Magnetic Resonance Imaging (MUMRI) Study

**DOI:** 10.1002/nbm.70347

**Published:** 2026-07-05

**Authors:** Ao Wang, Ian Schofield, Matthew G. Birkbeck, Daniel Baxter‐Beard, Andrew M. Blamire, Roger G. Whittaker

**Affiliations:** ^1^ Newcastle University, Translational and Clinical Research Institute (NUTCRI) Newcastle University Newcastle upon Tyne UK; ^2^ Northern Medical Physics and Clinical Engineering, Freeman Hospital Newcastle Hospitals NHS Foundation Trust Newcastle upon Tyne UK

**Keywords:** foot tapping, motor unit MRI, neuromuscular recruitment, rhythmic motor entrainment

## Abstract

Rhythmic motor paradigms are widely used to study sensorimotor timing, yet magnetic resonance imaging (MRI) research has largely focused on central processes, with limited insight into peripheral neuromuscular mechanisms. Motor unit MRI (MUMRI), a motion‐sensitive technique in which muscle contraction induces intravoxel water redistribution and transient signal attenuation, enables in vivo visualization of muscle activity. In this study, we developed and validated a combined behavioral–MUMRI paradigm to characterize muscle recruitment during rhythmic foot tapping. Healthy participants performed an auditory‐paced tapping task inside an MRI scanner while timing was recorded via an MRI‐compatible force transducer and muscle activity was measured using single‐slice MUMRI. A variable‐latency cueing design systematically sampled the temporal relationship between auditory cues, motor execution, and image acquisition, allowing identification of the optimal latency window for detecting contraction‐related signal changes. Fixed‐latency acquisitions were then used to assess reproducibility. Behavioral results showed stable performance across conditions, with low variability in tapping accuracy (mean coefficient of variation [CoV] ≈0.078). Transient, localized signal reductions consistent with muscle contraction were observed in anterior lower leg muscles during dorsiflexion. Voxel‐wise analyses demonstrated high within‐condition reproducibility and latency‐dependent spatial patterns, with the greatest average consistency when tapping aligned with scanner rhythm (*r* ≈0.68). These findings establish a robust framework for integrating rhythmic motor tasks with MUMRI, highlighting the importance of precise temporal alignment for reliable measurement of muscle activity. This approach provides a reproducible method for linking motor behavior to peripheral neuromuscular dynamics and offers potential for advancing both basic and clinical MRI research.

## Graphical abstract

We developed a reproducible motor unit MRI (MUMRI) foot‐tapping paradigm for imaging voluntary muscle recruitment during rhythmic movement. Healthy participants showed stable behavioral performance and consistent activation patterns. The protocol establishes an optimal acquisition strategy and supports future applications in neuromuscular disease research.

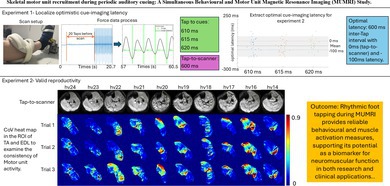

**Abbreviations**
ALS: Amyotrophic lateral sclerosis
CoV: Coefficient of variation
EDL: Extensor digitorum longus
EPI: Echo planar imaging
HV: Healthy volunteer
IRI: Inter‐response interval
ISI: Interstimulus interval
MRI: Magnetic resonance imaging
MU: Motor unit
MUMRI: Motor unit magnetic resonance imaging
PGSE: Pulsed gradient spin‐echo
PL: Peroneus longus
ROI: Region of interest
TA: Tibialis anterior
TR: Repetition time

## 1 Introduction

The ability to synchronize voluntary movements with external rhythmic cues, referred to as sensorimotor entrainment, is a fundamental feature of human motor control with both theoretical and clinical relevance. Rhythmic coordination supports activities ranging from speech and music performance to locomotion and relies on neural mechanisms that allow the brain to extract temporal regularities and predict the timing of upcoming events [1, 2]. Over the past decades, finger‐tapping paradigms have served as a widely used model for investigating the temporal coordination between perception and action. These studies have revealed a distributed neural network involving the basal ganglia, cerebellum, premotor cortex, and supplementary motor area that supports rhythmic motor timing and internal beat generation [3, 4]. They have also been instrumental in demonstrating how auditory rhythms facilitate movement by reducing temporal variability and enhancing phase‐locking between neural and motor activity [5].

Importantly, the potential of rhythm‐based paradigms has also been translated into clinical contexts. For example, auditory stimulation has been used to improve gait timing and step length in Parkinson's disease, highlighting the capacity of external cues to compensate for impaired internal timing mechanisms [6, 7]. Similar cue‐based approaches have been explored in stroke, cerebellar ataxia, and aging, suggesting that entrainment principles may serve as a generalizable framework for supporting motor rehabilitation [8, 9]. Despite these advances, most neuroimaging studies continue to rely on finger‐based tasks, due to their ease of implementation within the MRI environment and the minimal movement artifacts they produce. In contrast, lower limb movements such as stepping, marching, or foot tapping are critical for mobility and quality of life, but there are relatively few MRI studies in this area. Existing fMRI approaches using stepping or simulated movements often lack the temporal precision and muscle‐level detail needed to capture the neuromuscular mechanisms underpinning entrained leg movements [10]. Although fMRI provides valuable insights into cortical and subcortical processes associated with rhythmic movement, it does not provide a tool to study the peripheral mechanisms that actually generate the movement [3]. In the broader neuroimaging literature, positron emission tomography (PET‐MRI) has recently been used to investigate brain activity during walking [11], but such work likewise remains focused on central rather than peripheral mechanisms. To our knowledge, there are currently no MRI‐based studies that resolve muscle‐level activity patterns during rhythmic lower limb movements, underscoring the novelty of the present approach.

This gap is now addressable through the development of MUMRI, a motion‐sensitized imaging technique capable of detecting activity in individual or small clusters of motor units (MUs). MUMRI is based on the pulse‐gradient spin echo (PGSE) sequence and has already demonstrated its capability to resolve both single MU activity and large‐scale muscle recruitment, including in lower limb muscles [12, 13]. In MUMRI, brief muscle contractions induce localized, transient signal voids in the image due to microscopic intravoxel redistribution of water molecules during fiber shortening and relaxation [14]. These capabilities provide a unique opportunity to extend sensorimotor entrainment research beyond central timing networks and into the peripheral neuromuscular system, enabling simultaneous measurement of behavioral entrainment and its underlying muscle‐level implementation. Introducing this perspective is crucial to understanding the mechanisms that link central timing process to overt rhythmic movement, as rhythmic entrainment is not solely a central process but a distributed sensorimotor loop whose integrity depends on both predictive neural timing and efficient translation into organized MU output.

The present study integrates behavioral assessments of foot‐tapping accuracy with MUMRI recordings from lower leg muscles during a paced entrainment task. More broadly, this work aims to evaluate the feasibility of MUMRI as a noninvasive biomarker for early detection and longitudinal monitoring of neuromuscular impairment. Through this combined experimental and clinical perspective, we establish a conceptual progression from fundamental principles of rhythmic motor control to their translational relevance, ultimately positioning MUMRI‐based entrainment assessment as a clinically viable tool for neuromuscular diagnostics.

## 2 Methods

### 2.1 Participants

A total of 24 participants were recruited for this study; all participants were adults with right leg dominance. All participants provided written informed consent, and the study protocol was approved by the Newcastle University Faculty of Medical Sciences (FMS) Ethics Committee (reference number 2919/50852). Participants were recruited from the Newcastle University Centre of Transformative Neuroscience volunteer database via advertisement distributed through institutional mailing lists.

Participants reported no history of neurological or musculoskeletal disorders and reported normal hearing. All participants were right‐foot dominant.

### 2.2 MRI Data Acquisition

**FIGURE 1:**
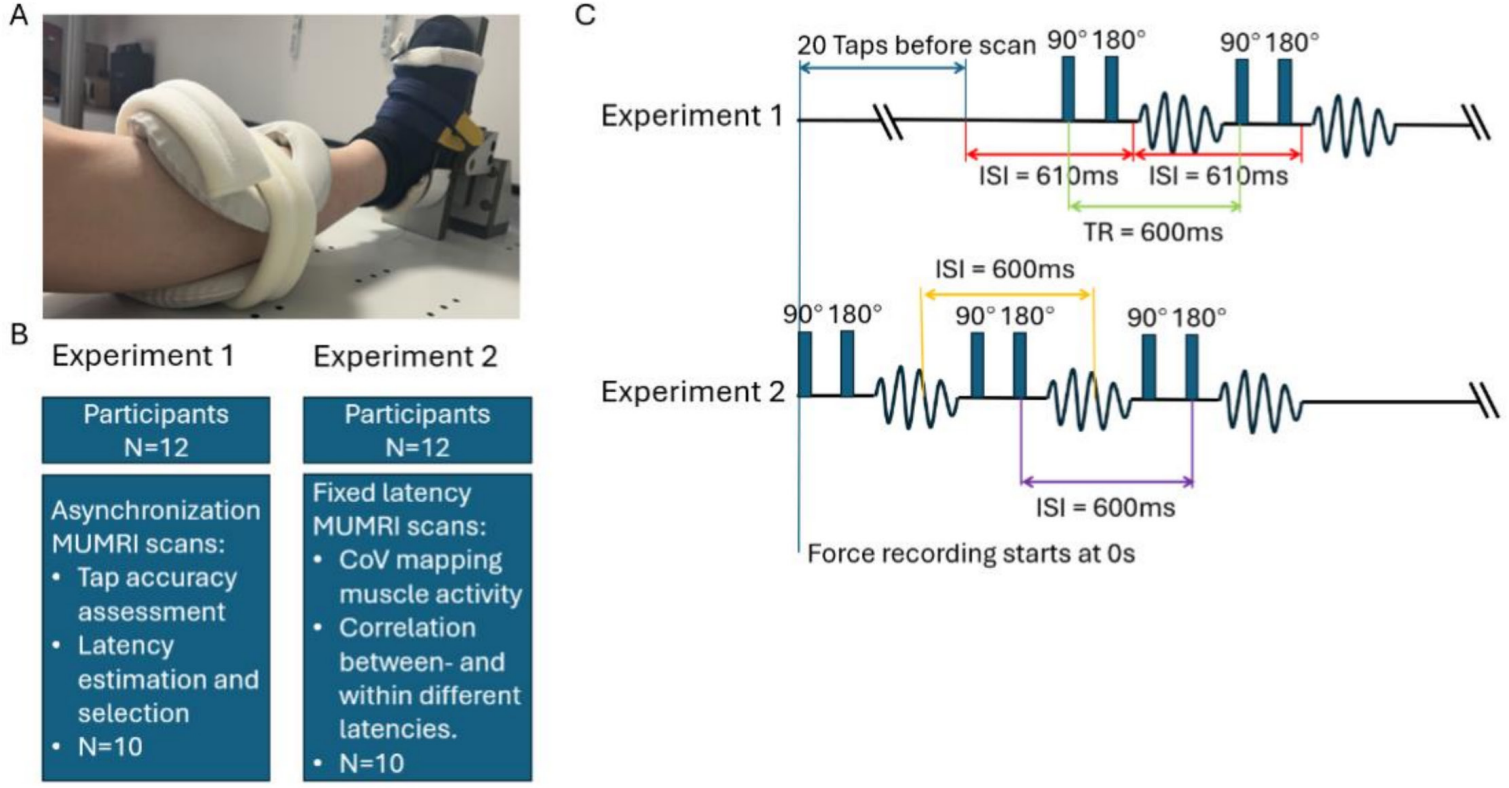
(A) MRI scanner with the custom‐built force plate. (B) Characteristic of the overall design of the experiments, two experiments do not share the same sample. (C) Schematic representation of the scan sequence and the designed Interstimulus interval (ISI). Blue line and arrows indicate the starting point of the force recording and the 20 taps before scanner initiation in Experiment 1. Red lines and arrows indicate the asynchronous cue intervals for the 21st and 22nd tap in a run of 610 ms condition in Experiment 1. Green lines and arrows indicate a TR of 600 ms in Experiment 1. Purple lines and arrows indicate a single tap‐to‐scan ISI. Yellow lines and arrows indicate a single −100 ms fixed‐latency ISI.

Participants lay supine in the MRI scanner with their right leg positioned in a relaxed, extended posture and their right foot securely strapped to a custom‐built immobile footrest incorporating a force transducer (Figure 1A; see also Experiment 1: Behavioral data Acquisition from The Force Transducer). They were instructed to perform the foot‐tapping task continuously during image acquisition, synchronizing their taps either to an audio cue delivered through MRI‐compatible headphones or to the intrinsic scanner noise.

Imaging was performed using a 3 T Philips Achieva system (Philips Healthcare, Best, The Netherlands). Images were acquired using a pair of 10‐cm elliptical flexible surface coils or a torso coil (Philips Medical Systems), positioned above and below the right calf muscle. Acquired images included a T1‐weighted structural scan, with the widest part of the calf selected for the single‐slice MUMRI.

MUMRI images collect a time series of scans repurposing the pulsed gradient spin‐echo (PGSE) preparation in a standard diffusion‐weighted imaging sequence with EPI readout (TR = 600 ms, TE = 40 ms, slice thickness = 8 mm, in‐plane resolution = 2 × 2 mm^2^) but using a very low motion sensitivity (b‐value = 1 s/mm^2^). Motion‐encoding gradients were applied along the slice‐select (through‐plane) direction only. Signal attenuation in MUMRI scans is driven by contraction‐induced intravoxel motion rather than diffusion processes. The magnitude of the motion is such that only a very low “*b*‐value” is required for efficient detection. Each MUMRI scan contained 120 dynamics (i.e., 120 consecutive image acquisitions at TR = 600 ms), corresponding to a total scan duration of approximately 72 s.

### 2.3 MRI Data Processing and Analysis

First, all image data were transferred from DICOM into NIfTI using the format conversion function of MRI‐specific software MRIcroGL, version 1.2.2 [15]. For each run, a single imaging slice was repeatedly acquired at each TR, resulting in a time series of serially collected images (hereafter referred to as “dynamics”). Images were then rigidly registered in‐plane to correct for potential drift during acquisition. Registration was performed using custom code written in MATLAB (version R2024b, MathWorks, Natick, MA, USA), ensuring spatial consistency across the time series.

On MUMRI scans, voluntary MU activity typically appears as localized, transient reductions in signal intensity that vary in size, shape, and timing across voxels, consistent with variable MU recruitment [12]. These signal dropouts arise because the PGSE sequence is sensitive to intravoxel motion: contraction of muscle fibers results in spatially heterogeneous deformation across the slice thickness, causing nonlinear rearrangement of water molecules that leads to signal loss [16]. The signal amplitude was visualized and measured using image processing software Fiji (ImageJ) within an ROI drawn confined for each participant to the tibialis anterior (TA) and extensor digitorum longus (EDL) [17]. These muscles were selected because they are primarily responsible for dorsiflexion. As the starting ankle angle was fixed at approximately 90° and because the tested foot was secured to the force transducer, the tapping movement was dominated by dorsiflexion.

### 2.4 Auditory Cues and Pre‐Scan Training

Two auditory cueing strategies were employed across the study. In Experiment 1, participants performed the task under externally cued conditions, in which brief tone pulses were delivered via MRI‐compatible headphones. These cues were presented at interstimulus intervals (ISIs) (610, 615, and 620 ms) that were slightly offset from the scanner repetition time (TR = 600 ms), thereby systematically varying the temporal relationship between auditory cueing, motor execution, and image acquisition. The primary aim of Experiment 1 was to characterize this temporal relationship and to identify the cue‐to‐scan latency that maximized the detectability of contraction‐related signal changes in the MUMRI data.

In addition to externally delivered cues, a scanner‐driven cueing condition (“tap‐to‐scanner”) was employed, in which participants synchronized their tapping to the intrinsic acoustic noise generated by the MRI sequence. This acoustic signal arises from periodic gradient switching during EPI readout and provides a highly regular temporal reference aligned with the TR.

In Experiment 2, a fixed‐latency design was implemented based on the temporal characteristics identified in Experiment 1. Auditory cues were delivered at a constant timing relative to the scanner TR to reproduce the selected cue‐to‐scan alignment, allowing evaluation of the reproducibility and stability of the resulting muscle activation patterns. The tap‐to‐scanner condition was also included in Experiment 2 to enable comparison between externally cued and scanner‐driven entrainment.

Prior to scanning, all participants underwent a standardized training procedure outside the scanner. This included a demonstration of the required tapping movement, presentation of audio examples corresponding to both cueing conditions, and live demonstration by the experimenter to ensure correct execution. Participants were instructed to perform rhythmic foot tapping in a relaxed and natural manner, without explicit requirements on force amplitude. Participants were specifically instructed to perform each tap by lifting the forefoot (dorsiflexion) followed by a relaxed return to the resting position, ensuring consistency in the movement pattern across participants. Tapping primarily involved dorsiflexion against the fixed footplate of the force transducer, resulting in constrained movement with limited joint displacement. Under these conditions, muscle contractions can be considered functionally quasi‐isometric, although participants were not explicitly instructed to maintain isometric force output. This prescan familiarization ensured that participants understood the timing requirements and could perform the task reliably before entering the MRI environment.

### 2.5 Experiment 1

To characterize the temporal relationship between rhythmic motor output and its expression in MUMRI, we implemented a two‐stage experimental design (Figure 1B). The first experiment employed an asynchronous paradigm to examine the precision of tapping in the MRI environment with behavioral data collected from a custom force recording system. This paradigm also determined the relative timing between the auditory‐paced cue, the executed movement, and the image acquisition that maximizes the detectability of motor unit evoked transient signal voids.

#### 2.5.1 Behavioral Data Acquisition From the Force Transducer

Participants lay supine in the MRI scanner with their right leg positioned in a relaxed, extended posture. The right foot was secured to a custom‐built rigid footrest incorporating a force transducer using non‐elastic straps placed across the forefoot and ankle, with foam padding to ensure comfort and minimize bulk movement. After the foot was secured, the force transducer was zero‐calibrated to account for baseline load and ensure accurate measurement of force fluctuations during the task.

The force transducer contained a binocular beam load cell (capacity 60 kg, resolution 3 g; Elane, China) [14]. The analog output of the load cell was amplified and digitized using purpose‐built MRI‐compatible electronics (designed in‐house) and transmitted via fiber‐optic cabling through a waveguide in the Faraday Cage to a data acquisition system in the control room. This ensured electrical isolation from the scanner environment, prevented RF interference, and allowed accurate synchronization between force measurements and image acquisition. Participants were instructed to tap their foot in synchrony with auditory cues delivered via MRI‐compatible headphones or with the intrinsic noise of the scanner (TR = 600 ms). Each tap consisted of a dorsiflexion (lift) followed by a plantarflexion (tap), with the plantarflexion phase aligned to the pacing cue. Each experiment was repeated three times to measure reproducibility.

The choice of an intersignal interval (ISI) of approximately 600 ms (610, 615, and 620 ms) was motivated by prior work on spontaneous motor tempo and rhythmic tapping behavior in healthy adults [18, 19]. Multiple studies have demonstrated that spontaneous finger‐tapping tempos remain highly stable across days and times of day, with mean intertap intervals typically clustering around the subsecond range and exhibiting relatively low intra‐individual variability [20, 21]. Importantly, tapping intervals in the vicinity of 600 ms fall within the range of naturally preferred motor tempos reported in reviewed studies, suggesting that this temporal scale aligns with intrinsic timing mechanisms of the motor system rather than imposing an artificial or cognitively demanding rhythm [22]. By adopting an intersignal interval close to this spontaneous motor tempo, the present experiment aimed to minimize excessive temporal variability and support reliable sensorimotor synchronization, thereby providing a physiologically and behaviorally valid temporal framework for assessing motor responses to auditory cues.

**FIGURE 2:**
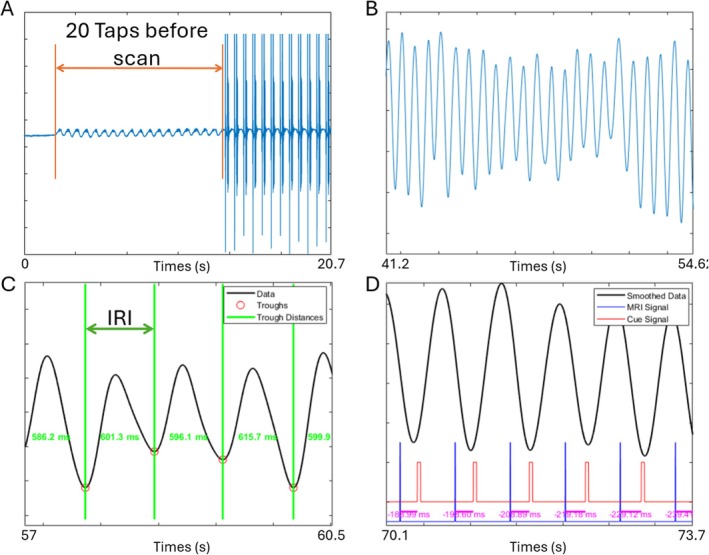
(A) Raw force signal (arbitrary units) collected from a participant tapping on the force transducer during scanning, where spikes in the plot were artifacts caused by the scanner image collection. Orange arrow indicates the 20 taps entraining before scanner initiation. (B) Processed signal after smoothing and filtering. (C) Annotated time series showing force trace (black), inter‐response interval (green), an example of IRI indicated with green arrow. (D) Asynchrony of cueing interval (red) and MRI image acquisition cycle (blue), pink numbers indicate the distance between the two, when it was minus zero the image acquisition is prior to the audio cue and when the audio cue delivered before image taken this number would be positive.

To quantify tapping behavior, MATLAB (version R2024b, MathWorks, Natick, MA, USA) was used to analyze the raw data collected from the force transducer. As shown in Figure 2A, the unprocessed force data consisted of voltage traces reflecting foot pressure over time. This signal was prone to artifacts caused by MRI sequence noise, which we addressed using a moving average smoothing followed by LOESS (locally estimated scatterplot smoothing) filtering. The cleaned data, shown in Figure 2B, allowed us to detect clear peaks and troughs corresponding to each foot tap. We identified tapping events by locating local troughs within the filtered signal (Figure 2C) and computed the inter‐response intervals (IRI) by measuring the time difference between consecutive taps. A time series of image acquisition, cue delivery, and tap events was produced for each run (Figure 2D).

Tapping accuracy was assessed in each participant by calculating the IRI and their variability. Two key metrics were derived: the mean of IRI errors and the coefficient of variation (CoV), computed as follows:

(1) $${\mathrm{IRI}}_{\text{error}}=\left(\mathrm{IRI}–\mathrm{ISI}\right)/\mathrm{ISI}\times 100\%.$$

(2) $$\text{Behavioral}\;\mathrm{CoV}=\left(\text{Standard Deviation of}\;\mathrm{IRI}/\text{Mean}\;\mathrm{IRI}\right)\times 100\%.$$

In these equations, IRI_error_ represents the percentage deviation of the tapping interval from the instructed stimulus–stimulus interval (ISI). The IRI_error_ metric is relevant because it quantifies how closely participant‐produced intervals align with the target pacing, thereby providing a direct measure of tapping timing accuracy, which is central to motor entrainment tasks. This metric has been widely used in tapping literature to distinguish systematic timing deviations from random variability in paced motor tasks [19].

To reduce the influence of extremely long intervals that likely reflect omitted or markedly delayed taps due to attentional lapses or transient disengagement, IRIs greater than 1.7 × IRI_median_ were classified as outliers. This threshold, adopted from the original paradigm, has been shown to effectively separate physiologically plausible timing variability from omission‐like events in paced motor tasks [19]. IRIs beyond this limit were excluded from the calculation of IRI_error_ and subsequent CoV analysis, meaning that only intervals considered representative of genuine rhythmic performance contribute to the accuracy metrics.

The removal affected only the distribution of IRIs used for computing accuracy and variability metrics of the force data. A negative mean of IRI_error_ indicated that the average interval between taps was shorter than the designated ISI for that session (e.g., a mean tapping interval of 602 ms in trials with a 610 ms target ISI would return an IRI_error_ of −0.33%). The behavioral CoV, reflecting the ratio of variability to the mean timing, served as an indicator of rhythmic motor control, with higher values indicating greater temporal inconsistency [23]. This metric was used to assess tapping stability and performance across experimental conditions and participants [1]. A behavioral CoV of 15% has been widely accepted in previous studies as the upper threshold for stable rhythmic performance [24]. We therefore used this value as a reference criterion for assessing tapping stability in the present study. Specifically, behavioral CoV values collected from each run of Experiment 1 were submitted to a one‐tailed, one‐sample *t*‐test against the 15% threshold to determine whether participants' tapping variability was significantly lower than the level typically associated with unstable or poorly entrained performance. In addition, behavioral CoV values were compared across ISI conditions using a one‐way ANOVA to assess whether tapping variability was influenced by cue timing or pacing source. These analyses allowed us to establish whether participants exhibited stable rhythmic entrainment under the experimental condition.

#### 2.5.2 Asynchronous Cue‐to‐Scan Design (Variable Latency)

Experiment 1 employed a variable‐latency design in which the auditory cue was intentionally offset from the scanner TR, causing the relative timing between cueing, tapping, and image acquisition to drift across the run. Auditory cues began 20 taps before the scan started, allowing participants to entrain themselves to the rhythm before data acquisition (Figure 1C). Each tapping block lasted 90 s and was repeated across three runs to provide sufficient data for both behavioral and MRI analyses.

Unlike the IRI measures described above, which quantify the temporal regularity of the tapping behavior, the MUMRI images allow us to visualize the specific muscles that are activated during each tap and to assess how reliably these muscle activation patterns are reproduced across repeated scans. In the lower leg, rhythmic dorsiflexion and plantarflexion primarily recruit the TA, EDL, and to a lesser extent, the medial and lateral gastrocnemius depending on the tap amplitude. MUMRI detects transient signal voids associated with individual or small clusters of MU twitches within these muscles, enabling direct observation of their spatial distribution [25].

By intentionally offsetting the auditory cue from the scanner TR, the cue‐to‐tap relationship drifted gradually over time, allowing multiple latencies to be sampled within a single run. This design enabled an empirical determination of the latency window during which the signal voids associated with MU activity were most consistently observed, thereby establishing the temporal constraints necessary for optimizing subsequent fixed‐latency acquisitions (Figure 2D).

To estimate the latency at which muscle activity was most effectively captured by the MUMRI acquisition, we quantified the temporal relationship between the auditory pacing cue and transient signal changes in MUMRI images.

**FIGURE 3:**
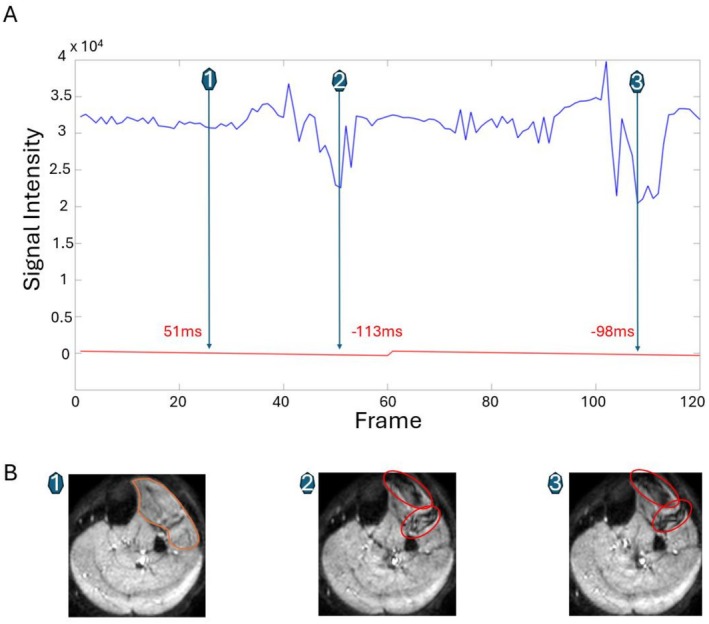
(A) An example of latency localization. The blue curve represents the signal intensity over the ROI of the TA and EDL area; the red curve shows the latency pattern resulting from the mismatch between auditory cues and scanner TR. The three pinpoints indicate the corresponding appearance on cross‐sectional images at each latency. (B) Examples showing the muscle activity of different frames. The orange shape indicates the region of interest; the first example also exhibits the cross‐section with no activity. Red circles in the second and third image indicate the voids that cause the decrease of the signal intensity. Numbers in red are the latency derived from each point.

For each run, the mean signal intensity across all voxels within the predefined muscle ROI of TA and EDL was computed for each of the 120 consecutive image frames, yielding a temporal intensity curve for that run (Figure 3A). Local troughs in this curve correspond to time points at which a maximal proportion of voxels exhibit contraction‐related signal loss and thus serve as an indirect marker of the temporal alignment between image acquisition and muscle activation (see Figure 3B).

For each run, we identified the three lowest troughs in the intensity curve. Selecting multiple troughs rather than a single minimum served two purposes: (i) to reduce the influence of noise or isolated artifactual drops in signal intensity and (ii) to ensure that the estimated latency reflected a reproducible physiological event rather than a single coincidence. These troughs were required to be separated by at least 20 image frames. This constraint ensured that the troughs correspond to distinct instances of cue‐tap‐scan alignment occurring at different phases of the progressive asynchrony between the cue interval (610, 615, or 620 ms) and the scanner TR (600 ms), which drifted systematically over the course of each run. A separation of 20 frames corresponds to approximately 12 s, encompassing multiple cycles of relative phase between cueing and scanning, and thus ensured that troughs were sampled from independent temporal alignments.

For each trough, the latency between the auditory cue and the corresponding image acquisition time point was computed based on the known cue timing and scanner trigger (Figure 3). The three latency values were then averaged across the three troughs to yield a representative optimal latency for that run. Run‐level estimates were then averaged across the three runs of each condition to obtain participant‐specific optimal latencies.

To characterize the overall temporal distribution, optimal latency estimates were subsequently pooled across participants. An empirical latency window was defined post hoc as the range within which the majority of optimal latencies fell. This distribution informed the selection of fixed‐latency conditions for Experiment 2.

### 2.6 Experiment 2

The second experiment then used a fixed‐latency design based on derived timing from *Experiment 1* to validate its robustness and to examine its generalizability across scanning conditions.

#### 2.6.1 Fixed Latency

Fixed‐latency conditions were generated by synchronizing the cueing system to the scanner TR. Scans were again acquired with TRs of 600 ms, with auditory cue timing adjusted to maintain the target latency in each condition. This approach allowed us to confirm that the previously identified latency consistently maximized the visibility of signal voids associated with MU activity.

Two tapping conditions were tested, referred to as “Tap to Scanner” and “−100 ms fixed latency” (Figure 1C). In the tap‐to‐scanner condition, participants synchronized their taps with the rhythmic sound of the MRI scanner itself rather than with an external auditory cue. The dominant component of this sound arises from rapid gradient coil switching, which produces a sharp, periodic acoustic pulse that can be readily perceived by participants. In the −100 ms fixed‐latency condition, participants were asked to ignore the scanner noise but focus on the auditory cue through the headphone, a similar process to Experiment 1 but with a single intercueing interval.

Each condition consisted of a 90 s tapping block, repeated three times to ensure adequate sampling of muscle activation patterns. There was a break after each three tapping scans to ameliorate the effect of fatigue. Unlike in the variable‐latency paradigm (Experiment 1), participants did not entrain 20 prescan taps before data acquisition but started to tap when the cue was delivered (e.g., either tap to the scanner noise or hear the cue from headphone).

#### 2.6.2 MRI CoV Analysis

To evaluate the stability and consistency of the MUMRI foot‐tapping paradigm, we performed a voxel‐wise analysis of signal variability across three repeated runs acquired under the same ISI or the same fixed latencies.

For both the tap‐to‐scanner and the fixed‐latency conditions, data were acquired using a single‐slice MUMRI sequence. For each voxel, the temporal mean, standard deviation, and signal range (max minus min) across the dynamic series were calculated. A voxel‐wise CoV map was then computed by dividing the temporal standard deviation by the mean signal intensity. In the context of MUMRI, MU activity manifests as transient signal voids, which produce increased temporal variability in voxel‐wise signal intensity. Voxels exhibiting higher CoV are interpreted as regions where contraction‐related signal fluctuations occur more frequently or with greater amplitude, consistent with active MU recruitment. Hence, CoV does not directly measure activation amplitude but rather reflects the temporal variability of signal intensity associated with repeated MU activity across the entire timeseries. To minimize contamination from nonmuscle tissues, noise, and edge effects, voxels were masked using intensity‐based thresholds determined individually for each run. Specifically, voxels with extremely low mean intensity (e.g., background or fat–air interfaces) or abnormally high signal values (typically corresponding to blood vessels) were excluded. The masking thresholds were defined relative to the signal distribution within the muscle ROI: Voxels whose mean intensity or CoV deviated by more than two standard deviations from the ROI average were excluded from further analysis. This statistical filtering removes extreme outlier voxels while preserving the majority of physiologically plausible muscle signal [26, 27]. It should be noted that the CoV used in the imaging analysis differs from the behavioral CoV described in Experiment 1. While behavioral CoV is expressed as a percentage to reflect variability in IRIs, the voxel‐wise CoV derived from MUMRI data is reported in fractional form, representing normalized signal variability across time.

For each tapping condition, CoV maps from repeated runs were compared to quantify within‐condition consistency. Pixelwise correlation was calculated between the repeated runs, yielding a correlation matrix that reflected how consistently the same physiological pattern was reproduced across runs of the same condition. Prior to statistical comparison, correlation coefficients were Fisher z‐transformed to improve normality and stabilize variance across the correlation range. All statistical analyses were performed on z‐transformed values. To assess the stability of the muscle activity pattern across different cue timings, a between‐group correlation analysis was also calculated. This produced a global correlation matrix representing the similarity of voxel‐wise temporal variability across conditions. The resulting correlation tables and representative range maps were saved for further visualization and statistical comparison.

## 3 Results

### 3.1 Sample Characteristics

Data from 20 participants were successfully acquired and included in the analysis (mean ± SD, age = 43.2 ± 13.9 years). Two participants' data were excluded in Experiment 1 due to a force transducer sampling rate failure, and two participants were unable to complete the protocol in Experiment 2 due to discomfort during scanning and voluntarily withdrew. Their data were removed from the results.

### 3.2 Experiment 1

#### 3.2.1 Behavioral Results

The mean CoV across all participants and conditions was 7.81% (SEM = 0.33%). A one‐tailed, one‐sample *t*‐test comparing foot‐tapping accuracy against a threshold CoV value of 15% revealed a statistically significant result, *t* (122) = −21.81, *p* < 0.001, indicating that the mean behavioral CoV across the participants was significantly lower than 15%. The one‐way ANOVA revealed that the individual behavioral CoV, which ranged from 4% to 12% (Table 1), was not significantly affected by the ISI, *F*(3, 122) = 0.794, *p* = 0.499, *η* ^2^ = 0.002. Details of behavioral CoV for each participant are shown in Table 1.

**TABLE 1:** Behavioral CoV in the reproducibility of the tap timing of participants for each targeted interstimulus interval in Experiment 1—Dyssynchronization trials.

Interval	610 ms	610 ms	610 ms	615 ms	615 ms	615 ms	620 ms	620 ms	620 ms	TR	TR	TR
Hv1	3.83	4.60	4.31	—	—	—	4.66	4.63	5.42	—	—	—
Hv2	6.77	4.96	5.99	—	—	—	4.65	4.63	4.70	—	—	—
Hv5	4.48	2.80	2.65	3.40	4.27	4.57	—	—	—	8.74	6.25	—
Hv6	7.94	6.90	6.82	7.51	7.48	11.22	9.72	8.81	12.26	—	—	—
Hv7	5.98	5.49	7.15	7.34	8.65	5.67	6.65	6.99	6.15	6.77	—	—
Hv8	10.67	9.61	10.97	9.30	10.99	10.25	10.23	10.76	10.28	8.84	10.25	11.64
Hv9	12.05	8.44	9.88	7.91	7.87	9.68	8.38	9.56	8.27	5.59	6.05	5.62
Hv10	6.53	5.63	4.66	4.90	5.02	6.55	4.49	4.64	5.42	5.18	4.49	5.26
Hv11	11.38	6.87	4.32	6.92	4.79	6.63	6.52	6.28	5.06	8.46	8.12	5.01
Hv12	3.82	5.49	4.77	4.33	5.03	4.42	5.63	5.21	5.56	7.31	2.84	3.54
**Mean**	**7.35**	**6.08**	**6.15**	**6.45**	**6.86**	**7.47**	**6.86**	**6.84**	**7.01**	**7.27**	**8.00**	**6.22**

*Note:* TR stands for tap to the scanner noise without external cueing through headphone, which has a cycle of 600 ms. All values are expressed as percentages. Blank cells (—) indicate conditions that were not acquired for all participants, as the final dyssynchronization protocol (610/615/620 ms) was implemented only after the initial phase of data collection.

Abbreviation: Hv, healthy volunteer.

#### 3.2.2 Latency Estimation and Selection

**FIGURE 4:**
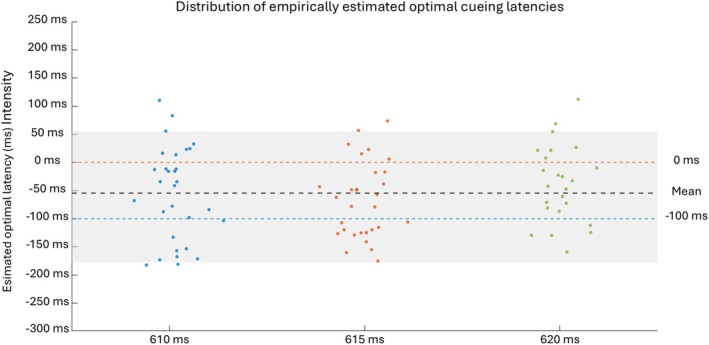
Distribution of empirically estimated optimal cue‐to‐scan latencies across participants in three different asynchronous cue‐to‐scan latencies. Each data point represents the average optimal latency estimated from a single test (run) for an individual participant. Negative values indicate that the peak muscle‐related signal change occurred prior to the image acquisition. The shaded gray region indicates the empirical latency window within which 95% of the averaged optimal latency falls in. Horizontal dashed lines denote the two fixed‐latency conditions selected for subsequent experiments: 0 ms (tap‐to‐scanner, synchronized to the intrinsic scanner rhythm) and −100 ms (auditory cue presented 100 ms prior to image acquisition) and the mean of all preserved latencies (−53.6 ms). The two fixed‐latencies were chosen to sample representative points within the empirically identified window while allowing direct comparison between scanner‐driven and externally cued entrainment.

As expected, interindividual variability was observed in the estimated optimal cue‐to‐scan latency (Figure 4). An optimized latency of 53.6 ms prior to the image acquisition was calculated (*μ* (84) = −53.61, SEM = 9.86).

Although the central tendency of the distribution was close to −50 ms, the majority of optimal latencies were distributed within a broader temporal window extending from approximately −180 to +50 ms relative to image acquisition (shade area in Figure 4). This range represents the period during which dorsiflexion‐related muscle activity most consistently produced detectable signal attenuation in the MUMRI sequence.

Based on this empirically derived latency window, two fixed reference latencies were selected for subsequent validation experiments. The first was 0 ms, corresponding to the tap‐to‐scanner condition in which participants synchronized their taps to the intrinsic acoustic rhythm of the MRI scanner. The second was −100 ms, representing an anticipatory cue‐to‐scan latency located within the empirically observed activation window and close to the midpoint of the negative‐latency range.

These two reference latencies were therefore chosen to sample distinct but representative points within the empirically identified activation window while also enabling a comparison between scanner‐driven synchronization and externally cued anticipatory tapping.

### 3.3 Experiment 2: CoV Analysis

**FIGURE 5:**
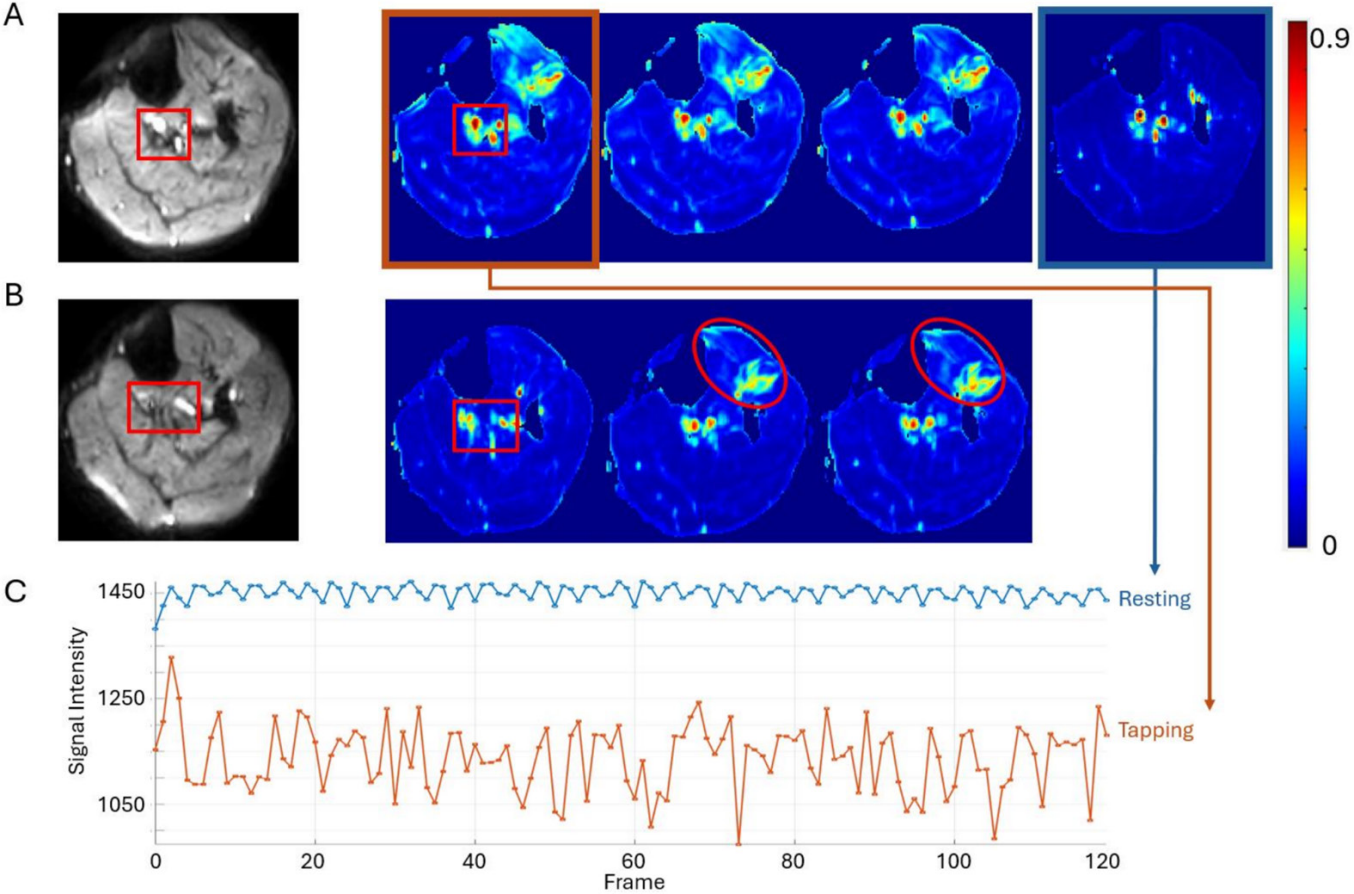
(A) The entire cross‐section view of CoV maps from the same participant tapping to the scanner, where the TA and EDL muscles show consistently high on motor unit activity pattern. (B) COV maps from the same participant tapping −100 ms before the image acquisition, where motor unit activity patterns of each run were inconsistently high from a whole cross‐section area viewing. Red squares indicate the cluster of vessels that appear as high CoV. Red circles indicate higher CoV appeared on the second and the third run but not the first run under the same condition. CoV was calculated as the standard deviation divided by the mean signal intensity (SD/mean) across the whole timeseries at each voxel, yielding a dimensionless measure of relative variability. Higher CoV indicates higher inconsistency of the MR signal, which implies more motor unit activity in the area. The color scale (0–0.9) represents increasing variability, with lower values indicating greater consistency across the time series (lesser motor unit activity). CoV values are expressed in fractional units (standard deviation divided by mean signal intensity) and are not directly comparable to behavioral CoV measures reported as percentages. (C) A line chart showing the alteration of the average signal intensity across the entire times‐series within the ROI of TA and EDL (see Figure 3, B1), blue line indicates the signal derived from a resting scan, which yield very low CoV, orange line indicates the signal derived from a tapping scan, the dramatically altering intensity reflects on CoV map as brighter color.

Qualitative inspection revealed that the spatial extent of MU activity varied systematically with latency, which was reflected in lower between‐group correlation from tap‐to‐scanner runs and −100 ms runs. However, this pattern was not uniformly observed across all participants. In some cases, the pattern of MU activity exhibited subtle differences across repetitions, while in others the amplitude or localization of the signal voids differed more markedly (Figure 5), note that CoV values reported here refer to voxel‐wise signal variability and are expressed in fractional units. These variations underscored the need for a quantitative metric capable of evaluating the degree of spatial reproducibility across repeated acquisitions. Therefore, to formally assess the stability of the MUMRI‐derived activation patterns within and between latency conditions, we computed voxel‐wise correlation matrices based on the CoV maps obtained from each run with the two fixed latencies: tap‐to‐scanner and −100 ms.

To illustrate these effects, Figure 5A,B shows whole cross‐sectional CoV maps from representative runs in one participant from the tap‐to‐scanner and −100 ms conditions, respectively. In the tap‐to‐scanner condition, the spatial pattern of signal voids within the anterior compartment, corresponding to the TA‐EDL region, appeared visually consistent across the three runs. By contrast, in the −100 ms condition, the first run displayed no anterior activity, whereas the second and third runs showed a broader and partially shifted activity in TA (red circle), indicating variability in spatial patterns across repetitions at this latency. Figure 5C provides a baseline comparison between the alteration of the average signal intensity from the resting scan and a tapping scan.

**FIGURE 6:**
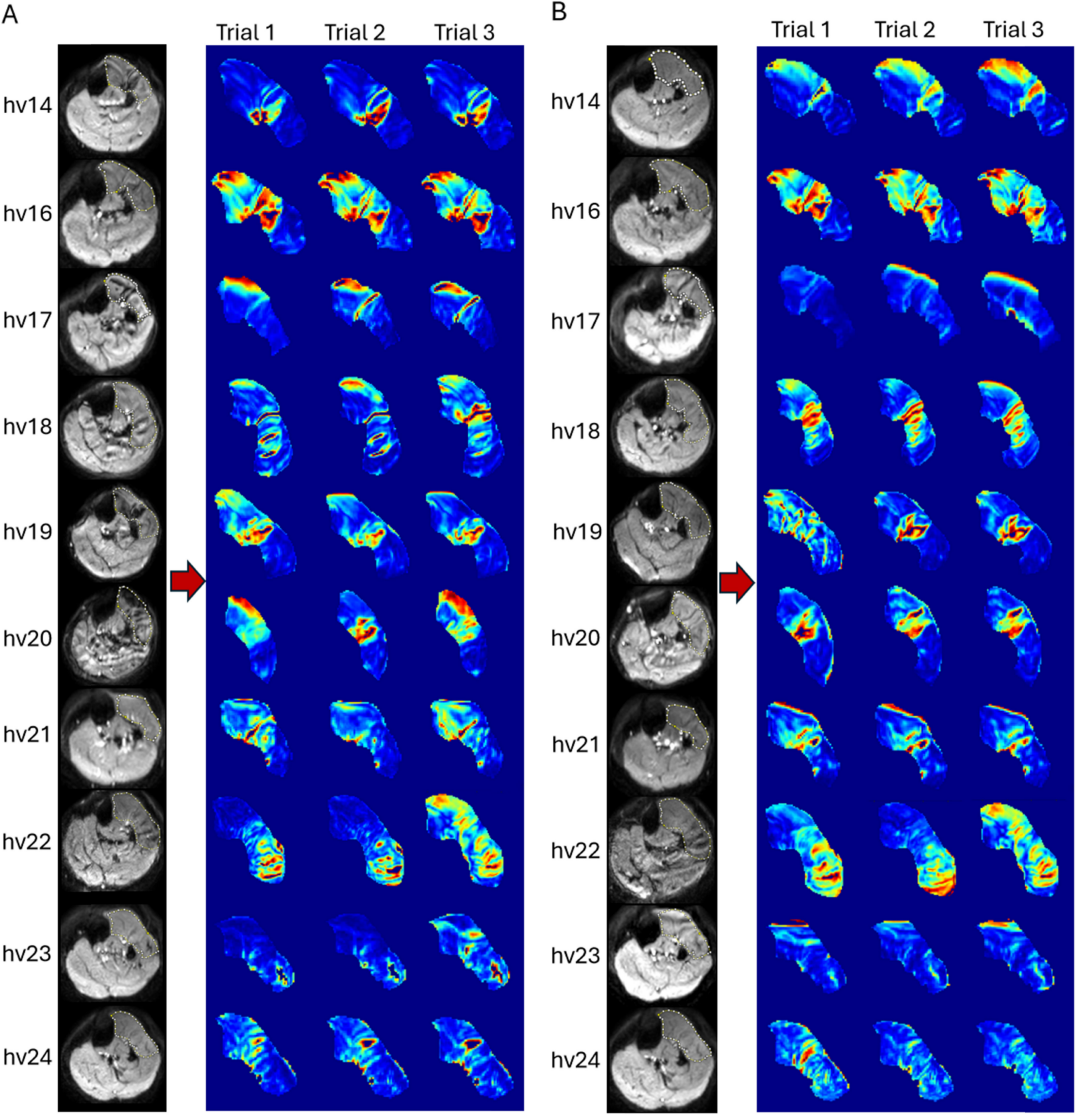
(A) CoV maps of three runs in tap‐to‐scanner trials for each participant. (B) CoV maps of three runs in 100 ms cue before image taking trials for each participant. Both conditions were ROI masked on TA and EDL regions. Hv 13 and 15 were excluded from the dataset due to failure to complete the protocol.

CoV maps for each of the participants undertaking three runs of the two conditions are shown in Figure 6. For the TR = 600 ms acquisitions, voxel‐wise CoV maps demonstrated good within‐condition reproducibility for both the tap‐to‐scanner (Figure 6A) and −100 ms (Figure 6B) conditions. After Fisher's *z* transformation of the correlation coefficients between repeated runs, the tap‐to‐scanner condition showed a mean within‐condition consistency of *z* = 0.84 (SD = 0.35), corresponding to a correlation of approximately *r* ≈0.68. The −100 ms condition yielded a slightly lower, but still robust, within‐condition mean of *z* = 0.66 (SD = 0.39; *r* ≈0.58).

Between‐condition correlations, computed between tap‐to‐scanner and −100 ms CoV maps, resulted in a mean Fisher's *z* of 0.63 (SD = 0.14; *r* ≈0.56). Thus, both paradigms produced broadly similar spatial patterns of temporal variability but within‐condition correlations were higher than between‐condition correlations, and this effect was most pronounced for the tap‐to‐scanner runs. These findings indicate that, at TR = 600 ms, the MUMRI foot‐tapping paradigm yields internally consistent voxel‐wise activation patterns, with each tapping condition showing greater reproducibility within itself than across different timing conditions.

**FIGURE 7:**
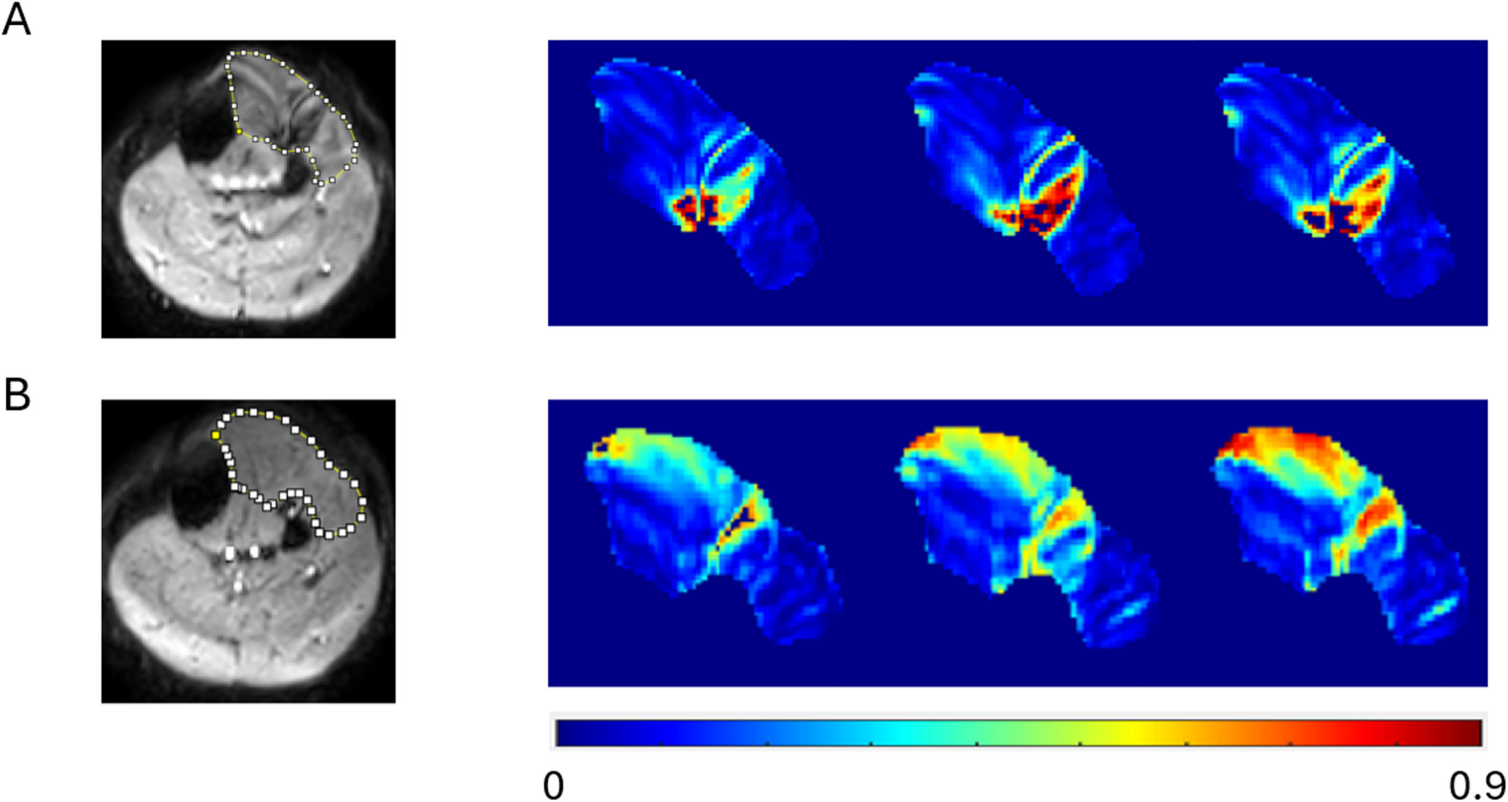
(A) Case from one participant tapping to the scanner, where the EDL muscles show high consistency on motor unit activity pattern from a CoV map of the ROI. (B) Case from one participant tapping −100 ms before the image acquisition, where motor unit activity patterns of each run differed and was mainly concentrated in the head of TA. CoV values are expressed in fractional units (standard deviation divided by mean signal intensity) and are not directly comparable to behavioral CoV measures reported as percentages.

Taken from the results above, a representative example from one participant further illustrates the latency‐dependent differences in muscle activation patterns (Figure 7). In the tap‐to‐scanner condition (Figure 7A), the spatial distribution of motor unit‐related signal voids was concentrated primarily in the region between the TA and EDL, forming a stable and well‐localized band of activity across repeated runs. By contrast, in the −100 ms condition (Figure 7B), activation shifted superiorly, with the strongest signal occurring in the upper portion of the TA and reduced involvement of the TA–EDL interface while losing consistency.

## 4 Discussion

Our study investigated the feasibility of using MUMRI to characterize MU activity during an auditory‐paced foot‐tapping task. Across both the variable‐latency and fixed‐latency paradigms, our findings demonstrate that rhythmic foot tapping can be performed with high temporal precision within the MRI environment and that the resulting MUMRI signal contains reproducible spatial structure that reflects underlying muscle recruitment patterns. These results position MUMRI‐based rhythmic paradigms as a promising approach for probing neuromuscular function in both basic and clinical research.

Participants showed highly consistent tapping performance across all auditory cueing intervals, with average CoV values (mean = 7.81%, SEM = 0.33%) significantly below the widely accepted 15% stability threshold [5, 19]. Prior work has shown that scanner noise can interfere with auditory perception and timing [28, 29], yet participants in this study maintained precise rhythmic synchronization after proper instructions, suggesting that predictive timing mechanisms remain robust under such conditions. Behavioral stability is particularly important in the context of MUMRI, as it ensures that cycle‐to‐cycle variability in the MRI signal reflects neuromuscular dynamics rather than irregularities in motor timing. The clean force traces and regular interval timing observed in the behavioral data therefore establish a reliable foundation for interpreting the muscle‐level information captured in the imaging data.

The asynchronous paradigm provided a systematic means of determining the temporal alignment between neuromuscular contraction and image acquisition that maximizes the visibility of motor unit‐related signal voids. By intentionally desynchronizing the cue interval from the TR, the relative timing of these events drifted across each run, enabling a full sampling of cue‐to‐scan latencies (Figure 4). Analysis of the temporal intensity curves revealed a broad latency window, extending from approximately −180 to +50 ms relative to the onset of image acquisition, within which muscle‐evoked signal decreases were most reliably detected (Figure 5). The central tendency of the optimal latency distribution was approximately −50 ms, indicating that presenting the cue shortly before image acquisition maximized the likelihood that the scanner captured the peak contraction‐related signal change.

Identifying this empirical activation window was critical for designing the fixed‐latency validation experiments. Rather than selecting a single optimal latency, we chose two representative reference points within this window: −100 ms, corresponding to an anticipatory cue‐to‐scan offset preceding the expected contraction peak, and 0 ms, corresponding to synchronization with the intrinsic acoustic rhythm of the MRI scanner. This design allowed us to sample different temporal positions within the activation window while also comparing externally cued anticipatory tapping with scanner‐driven rhythmic entrainment.

The presence of optimal latencies extending slightly beyond the nominal image acquisition time point (up to ~50 ms of postacquisition) likely reflects predictive motor timing rather than purely reactive responses to the auditory cue. In rhythmic synchronization tasks, participants typically exhibit a well‐documented phenomenon known as negative mean asynchrony, whereby motor responses precede the pacing stimulus by several tens of milliseconds [30, 31]. This anticipatory behavior is thought to arise from predictive internal models of temporal regularity rather than stimulus‐driven reaction. Under such conditions, muscle activation may be initiated prior to the nominal cue onset, resulting in contraction‐related signal changes that overlap with image acquisition even when the cue‐to‐scan latency appears slightly positive. Similar anticipatory effects have been reported extensively in sensorimotor synchronization literature, where taps reliably precede auditory beats despite explicit instructions to synchronize with them.

In the fixed‐latency experiments, voxel‐wise CoV maps demonstrated substantial reproducibility of MU activation. At TR = 600 ms, the tap‐to‐scanner condition yielded particularly high within‐condition correlations, indicating that synchronizing tapping to the intrinsic noise of the scanner produces stable spatial signatures of muscle activation variability. The −100 ms latency condition also showed good reproducibility, although slightly lower than the tap‐to‐scanner condition, suggesting that tapping to a continuous, salient, and regular acoustic cue (i.e., scanner noise) may reduce uncertainty relative to externally delivered but more complex auditory cues. This may reflect the fact that the high‐intensity, continuous acoustic background of the scanner elevates the baseline activity of the auditory system and reduces perceptual sensitivity to externally presented cues, thereby altering how timing of the taps is processed [29]. In both cases, within‐condition correlations exceeded between‐condition correlations, demonstrating that CoV maps capture condition‐specific neuromuscular signatures. This pattern was visually evident in the CoV maps, which exhibited consistent spatial structures across repeated runs within each condition but differed systematically between conditions (Figure 6).

The reproducibility of CoV maps under fixed latency indicates that the muscle activation patterns elicited by rhythmic foot tapping are inherently stable across repeated cycles and that MUMRI can capture these stable neuromuscular signatures with high fidelity (Figure 7). This reliability is critical for future applications of the method in both research and clinical contexts. The repeatability of spatial activation patterns suggests consistent recruitment of muscle compartments and MUs during rhythmic dorsiflexion. During tapping, plantarflexor muscles such as the gastrocnemius and soleus generate the primary force during the downward phase of the movement, whereas dorsiflexor muscles such as the TA and peroneal group are predominantly active during the upward phase. Because the force transducer system in the present study limited the angle of plantarflexion, the MUMRI data primarily reflect dorsiflexor‐related neuromuscular activity. The stability of these spatial patterns implies that MU territories, recruitment order, and coordination strategies remain consistent within individuals. Differences in consistency across cueing conditions further suggest that entrainment modality can influence recruitment heterogeneity, thereby providing insight into how central timing cues are translated into peripheral muscle activation.

The combination of behavioral entrainment measures and MUMRI‐based neuromuscular signatures has important translational implications. To date, most investigations of single MU activity using MRI have relied on electrical stimulation evoked contractions, in which peripheral nerves are stimulated to elicit isolated MU responses [25]. While such paradigms provide precise experimental control, they are experimentally constrained and may be uncomfortable, limiting their applicability in broader or more vulnerable populations.

In contrast, the present study demonstrates that reproducible MU–related signal patterns can be captured during voluntary, rhythmically entrained movement without the need for electrical stimulation. This represents a shift toward a more physiologically relevant framework for studying MU recruitment, preserving the natural coupling between central timing processes and peripheral muscle activation. By embedding neuromuscular imaging within a controlled yet functional motor task, the paradigm captures recruitment dynamics under conditions that more closely approximate natural movement.

Behavioral metrics assess peripheral sensorimotor synchronization, whereas voxel‐wise CoV maps provide insight into neuromuscular stability and recruitment coherence. Together, these complementary measures form a multidimensional assessment framework that may facilitate future translational applications, including the investigation of neuromuscular disorders. Disorders characterized by altered MU recruitment, such as motor neurone disease or peripheral neuropathies, may particularly benefit from a noninvasive, voluntary task‐based imaging approach [32, 33]. The present work therefore extends MUMRI beyond electrically evoked paradigms and establishes a foundation for studying human MU function under voluntary movement conditions.

Several limitations warrant consideration. First, the dyssynchronization parameters were refined during an initial pilot phase, such that not all latency conditions were acquired for all participants. While this staged implementation allowed optimization of the paradigm, it resulted in incomplete sampling of some conditions across the full cohort. Then, the use of a single‐slice MUMRI acquisition restricts spatial coverage and may fail to capture broader patterns of activation across synergistic muscle groups. In addition, the deliberate selection of an imaging latency aligned with the dorsiflexion phase necessarily biases the measurements toward dorsiflexor activity and limits the ability to characterize plantarflexor recruitment during the downward phase of the movement. Employing multislice or volumetric acquisitions in future studies would enable more comprehensive mapping of leg muscle dynamics. Additionally, while reproducibility was demonstrated in healthy participants, validation in larger clinical cohorts is needed to assess the sensitivity of CoV metrics to pathological neuromuscular changes. Exploring rate‐dependent modulation, fatigue effects, and more complex movement patterns may further enhance the sensitivity and interpretive power of the paradigm.

In conclusion, the present study demonstrates that rhythmic foot tapping during MUMRI yields stable behavioral timing and reproducible voxel‐wise muscle activation patterns across controlled cueing conditions. The integration of behavioral synchrony, latency estimation, and CoV‐based neuromuscular metrics provides a powerful approach for probing the dynamics of muscle recruitment. These findings establish a solid foundation for using MUMRI derived measures as biomarkers of neuromuscular function and support the broader potential of rhythmic motor paradigms in both fundamental motor control research and clinical applications.

## Author Contributions

**Ao Wang:** conceptualization, investigation, writing – original draft, methodology, writing – review and editing, formal analysis, project administration, and data curation. **Ian Schofield:** conceptualization, software, investigation. **Matthew G. Birkbeck:** conceptualization, methodology, writing – review and editing. **Daniel Baxter‐Beard:** investigation. **Andrew M. Blamire:** conceptualization, investigation, funding acquisition, writing – review and editing, supervision. **Roger G. Whittaker:** conceptualization, investigation, funding acquisition, writing – review and editing, project administration, supervision, and resources.

## Funding

This work was funded by the MND Scotland (2023/MNDS/6300/740WHIT). MND Scotland is the leading Scottish charity dedicated to supporting people affected by Motor Neurone Disease (MND), funding research for cures, and campaigning for improved care. They provide financial grants, counselling, and welfare advice while funding clinical trials and promoting awareness to find a cure. The views expressed are those of the author(s) and not necessarily those of MND Scotland.

## Conflicts of Interest

The authors declare no conflicts of interest.

## Data Availability

The data that support the findings of this study are available on request from the corresponding author. The data are not publicly available due to privacy or ethical restrictions.
